# Retinal microvascular and neuroretinal structural changes in pulmonary arterial hypertension assessed by OCTA: a comparative study

**DOI:** 10.55730/1300-0144.6181

**Published:** 2026-01-30

**Authors:** Murat ERDAĞ, Tunahan ÇALIŞKAN, Hakan YILDIRIM, Tarık KIVRAK, Mehmet CANLEBLEBİCİ, Ali DAL, Mehmet BALBABA

**Affiliations:** 1 Department of Ophthalmology, Faculty of Medicine, Fırat University, Elazığ, Turkiye; 2 Department of Cardiology, Faculty of Medicine, Fırat University, Elazığ, Turkiye; 3 Department of Ophthalmology, Kayseri State Hospital, Kayseri, Turkiye; 4 Department of Ophthalmology, Faculty of Medicine, Mustafa Kemal University, Hatay, Turkiye

**Keywords:** Pulmonary arterial hypertension, optical coherence tomography angiography, retinal microvasculature, vascular density, systemic microvasculopathy

## Abstract

**Background/aim:**

Pulmonary arterial hypertension (PAH) is a progressive disorder with systemic vasculopathy, and noninvasive assessment remains challenging. The retina, sharing anatomical similarities with the cerebral microcirculation, is an accessible site for visualizing systemic vascular compromise. This study quantitatively evaluated retinal microvascular and neuroretinal structural changes in PAH patients compared to healthy controls using optical coherence tomography angiography (OCTA).

**Materials and methods:**

In this single-center, cross-sectional case-control study, 40 patients with group 1 PAH and 38 healthy controls were enrolled. For statistical independence, only the right eye of each participant was analyzed (total: 78 eyes). All participants underwent high-resolution OCTA imaging. Key metrics including vascular density (VD) in the superficial (SCP) and deep capillary plexus (DCP), foveal avascular zone (FAZ) flow density (FD), and neuroretinal thicknesses (ganglion cell complex [GCC] and retinal nerve fiber layer [RNFL]) were quantified and compared using nonparametric tests.

**Results:**

Comparison of PAH patients and controls revealed significantly reduced VD in the PAH group. In the SCP, VD was significantly lower in the whole image (p = 0.004) and the ETDRS grid (p = 0.005). Similarly, the DCP showed reduced VD in the whole image (p = 0.006). Quadrant analysis indicated marked reduction in the superior quadrant for both plexuses (p < 0.01). While FAZ area was similar between the groups (p = 0.779), FAZ FD was significantly decreased in PAH patients (p = 0.001). Structurally, mean GCC thickness was significantly reduced (p = 0.006), but RNFL thickness remained preserved (p = 0.267).

**Conclusion:**

PAH is associated with significant retinal microvascular density reduction across all plexuses. These findings provide evidence corroborating the systemic nature of PAH vasculopathy. OCTA is a promising noninvasive tool for assessing systemic microvascular involvement in this population. However, further longitudinal studies are needed to validate its utility for disease monitoring.

## 1. Introduction

Pulmonary arterial hypertension (PAH) is a progressive and life-threatening vasculopathy characterized by precapillary pulmonary hypertension and substantial morbidity and mortality [1,2]. Although traditionally regarded as a pulmonary-limited disease, accumulating evidence indicates that PAH is accompanied by systemic endothelial dysfunction, affecting multiple vascular beds beyond the lungs [3–5]. Given its shared embryological origin, autoregulatory function, and microvascular architecture, the retina offers an accessible and noninvasive means to evaluate systemic microcirculatory health [5].

Ocular manifestations of PAH including increased retinal venous tortuosity, choroidal congestion, and serous retinal detachment reflect the combined effects of elevated central venous pressure, hemodynamic burden, and neurovascular dysregulation [6–9]. However, current diagnostic and follow-up strategies rely predominantly on right heart catheterization, which is invasive and unsuitable for repeated assessments [10]. Echocardiography, though widely used, has well-recognized limitations in detecting subtle microvascular impairment [4,11]. This clinical gap underscores the need for reliable, noninvasive biomarkers capable of tracking systemic microvascular involvement and improving disease monitoring.

Optical coherence tomography angiography (OCTA) offers high-resolution, depth-resolved visualization of the retinal microvasculature, enabling quantitative assessment of vessel density (VD), foveal avascular zone (FAZ) metrics, and neuroretinal structural integrity [12–14]. Emerging studies have reported reduced retinal VD and thinning of inner retinal layers such as the ganglion cell complex (GCC) and retinal nerve fiber layer (RNFL) in PAH [15,16]. However, previous investigations were limited by small sample sizes, heterogeneous patient groups, and incomplete exclusion of retinal comorbidities. Furthermore, whether OCTA-derived measures consistently reflect systemic microvascular involvement in well-defined populations with group 1 PAH remains insufficiently explored.

The present study aims to quantitatively assess retinal microvascular and neuroretinal structural changes in a rigorously characterized cohort of patients with group 1 PAH compared to healthy controls. By employing strict ocular exclusion criteria and standardized high-quality imaging parameters, we sought to determine whether OCTA-derived metrics can function as robust, noninvasive indicators of PAH-associated systemic microvascular compromise.

## 2. Materials and methods

This single-center, cross-sectional, case-control study was conducted in accordance with the Declaration of Helsinki. The study protocol was approved by the institutional review board of the participating institution (Approval No: 2025/16-32) and written informed consent was obtained from all participants prior to enrollment. Eligible participants were prospectively recruited between March 2025 and October 2025 from among patients under follow-up in a tertiary cardiology clinic. Individuals with a confirmed diagnosis of group 1 PAH, as defined by the World Health Organization classification, were included [17].

The inclusion criteria required participants to be 18 years of age or older and to have a confirmed diagnosis of PAH based on established hemodynamic criteria, specifically including mean pulmonary arterial pressure of ≥20 mmHg as measured by right heart catheterization. Exclusion criteria were rigorously applied to preserve the validity of the ophthalmic measurements. Patients with known glaucoma, uveitis, diabetic retinopathy, age-related macular degeneration, or any other retinal pathology were excluded. Additional exclusion criteria included the presence of significant cataract or corneal opacities that could compromise image quality, as well as a history of ocular surgery or ocular trauma.

To ensure comparability and eliminate potential confounders affecting OCTA metrics, all participants, including those in the control group, underwent a comprehensive ophthalmic evaluation that included best-corrected visual acuity assessment, intraocular pressure measurement, slit-lamp biomicroscopy, dilated fundus examination, and autorefractometry to document spherical equivalent refraction. These parameters were reviewed to confirm the absence of exclusion criteria and to verify that refractive errors, media clarity, or ocular pressure variations that might influence vascular measurements were not significantly different between the groups.

The control group consisted of healthy age- and sex-matched individuals with no history of systemic or ocular disease and with normal findings on complete ophthalmic examination. For all PAH patients, relevant clinical data were obtained from medical records prior to ocular imaging.

Ophthalmic imaging was subsequently performed using the Optovue Solix device (Optovue, Fremont, CA, USA). All OCTA images were acquired using a standardized protocol by trained personnel, and only high-quality scans meeting predefined quality criteria were included in the analysis. These criteria included a signal strength index (SSI) value of ≥6 and a lack of motion or segmentation artifacts. Quantitative parameters derived from the OCTA and optical coherence tomography (OCT) scans included VD for both the superficial capillary plexus (SCP) and the deep capillary plexus (DCP), FAZ metrics (area and flow density [FD]), and the neuroretinal thickness parameters of mean GCC and RNFL. To ensure statistical independence and avoid potential bias arising from interocular correlation, only the right eye of each participant was assessed and included in the analysis.

Statistical analysis was conducted using IBM SPSS Statistics 31 for Mac (IBM Corp., Armonk, NY, USA). Descriptive statistics were presented as mean and standard deviation (SD). The Shapiro–Wilk test was used to assess the normality of the data (p < 0.05). The Mann–Whitney U test was employed to compare all OCTA and retinal parameters between two independent groups (i.e., PAH patients vs. healthy control group), as the data distribution did not consistently meet the criteria for parametric testing. A p-value of less than 0.05 was defined as statistically significant for all comparisons. Given the exploratory nature of the study and the limited sample size, no correction for multiple comparisons was applied to avoid excessive type II error.

## 3. Results

### 3.1. Baseline characteristics of the study population

The study successfully enrolled 40 patients with group 1 PAH and 38 healthy control subjects. The groups were statistically comparable regarding critical demographic factors, showing no significant differences in mean age (55.55 years vs. 50.42 years; p = 0.180) or sex distribution (p = 0.059). The mean best-corrected visual acuity was 0.75 ± 0.25 in the PAH group, comparable to that of the control group (p > 0.05). Intraocular pressure measurements were within normal limits for both groups (PAH: 14.78 ± 2.25 mmHg vs. control: 14.50 ± 2.10 mmHg; p > 0.05). To rule out the magnification effect of refractive error on OCTA measurements, spherical equivalent refraction was confirmed to be statistically similar between the PAH group (+0.27 ± 1.30 D) and the control group (+0.25 ± 1.15 D; p > 0.05), as seen in Table 1.

### 3.2. Comparison of OCTA and structural OCT parameters

A comprehensive comparison of microvascular and neuroretinal parameters between the PAH patients and the control group revealed evidence of ocular compromise, as summarized in Tables 2–5.

#### 3.2.1. Retinal microvascular impairment (OCTA)

Statistically significant reductions in VD were observed across multiple layers of the retina in the PAH group compared to the control group. In the SCP, significant differences were found in mean VD for the whole image (p = 0.004) and for the Early Treatment Diabetic Retinopathy Study (ETDRS) grid (p = 0.005), as shown in Table 2. This microvascular deficit was similarly pronounced for the DCP, where the mean VD for the whole image (p = 0.006) and for the ETDRS grid (p = 0.008) was significantly lower in PAH patients (Table 3).

In quadrant analysis, statistically significant reductions were observed in the superior quadrant for both the SCP (p = 0.006, Table 2) and the DCP (p < 0.001, Table 3). However, the results for the inferior quadrant differed between the layers; while the SCP showed a significant reduction (p = 0.020, Table 2), the reduction in the DCP did not reach the level of statistical significance (p = 0.074, Table 3).

Furthermore, analysis of the FAZ metrics revealed that although the FAZ area itself did not show a significant difference (p = 0.779, Table 4), the density of the surrounding microvasculature, quantified by the FAZ FD, was markedly decreased in the PAH group (p = 0.001, Table 4).

#### 3.2.2. Neuroretinal structural OCT changes

As detailed in Table 5, the mean GCC thickness was significantly reduced in the PAH group compared to the control group (p = 0.006), suggesting neuroretinal vulnerability. In contrast, the other structural parameters listed in Table 5, including mean RNFL thickness (p = 0.267), foveal thickness (p = 0.749), and disc area (p = 0.686), remained statistically similar between the two groups.

## 4. Discussion

In this cross-sectional case-control study, retinal microvascular and neuroretinal structural changes in patients with PAH were quantitatively evaluated using OCTA. Our findings demonstrate a significant reduction in vascular density within both the superficial and deep retinal capillary plexuses, accompanied by macular capillary hypoperfusion. These results indicate that the microvascular involvement observed in PAH is also detectable at the retinal level, supporting the hypothesis that OCTA-derived parameters can reflect systemic vascular impairment.

The core findings of our study indicate a significant reduction in retinal vascular density across both the SCP and DCP together with a marked decrease in FAZ FD in the PAH group. These findings are strong evidence of the systemic vasculopathy associated with PAH being extensively propagated to the retinal microcirculation. In leveraging the capacity of OCTA for noninvasive, layer-specific vascular assessment [18], these findings corroborate the hypothesis that PAH possesses a systemic microvasculopathy component and that the eye may serve as a noninvasive “window” reflecting those systemic alterations.

It is increasingly acknowledged that PAH extends beyond the pulmonary vasculature to affect extrapulmonary systems, potentially including the ocular circulation, through mechanisms involving elevated systemic venous pressure [6], systemic inflammation [19], and endothelium-derived mediators [20]. Increased superior vena cava pressure elevates ophthalmic vein pressure, inducing ocular venous dilation and choroidal congestion [21], which consequently leads to stasis within the ocular capillary network [22]. Our principal findings demonstrate a significant reduction in VD within both the SCP and DCP, as well as a decrease in the FAZ FD. Furthermore, our data indicate a topographic disparity: vascular depletion was markedly more severe in the superior regions compared to the inferior regions. The lack of statistical significance in the inferior DCP despite global reduction suggests that vascular damage in PAH may begin sectorally rather than uniformly, potentially making the superior quadrant a more sensitive site for early screening.

The widespread and significant reduction in both SCP and DCP VD observed in our study closely mirrors previously reported OCTA findings in PAH patients. Earlier work, including the study by Gu et al. [15], used different grouping strategies such as stratifying patients according to fundus abnormalities, while we excluded all preexisting retinal pathologies and evaluated PAH cases as a single homogeneous cohort. However, the convergence of results across such methodological differences supports retinal microvascular compromise as a reproducible feature of PAH. Importantly, this pattern is not exclusive to PAH; similar reductions in retinal VD have been documented in systemic hypertension [23], coronary artery disease (CAD) [24], and congestive heart failure [25]. Wang et al. [26] further demonstrated that increasing CAD severity correlates with straighter retinal arterioles and narrower venular branching angles, suggesting that systemic cardiovascular dysfunction can influence not only perfusion metrics but also microvascular geometry. While some cardiovascular diseases appear to preferentially affect deeper vascular layers, PAH demonstrates a more diffuse involvement extending across both superficial and deep plexuses. This broader pattern aligns with evidence from extracutaneous microcirculation showing heightened vasoconstrictor tone and diminished vasodilatory reserve in idiopathic PAH compared to chronic thromboembolic pulmonary hypertension [27]. Collectively, these findings help to confirm that OCTA-detectable capillary rarefaction reflects a systemic microvascular dysfunction in PAH rather than an isolated ocular phenomenon.

The underlying mechanisms driving this vascular impairment are likely a confluence of hemodynamic and cellular/molecular factors. Venous stasis and choroidal congestion constitute fundamental hemodynamic contributors [28]. At the cellular level, endothelial dysfunction, systemic inflammation, and immune dysregulation may exert significant influence. Endothelial injury can activate adventitial fibroblasts, thereby contributing to vascular remodeling [29]. The presence of autoantibodies such as antiendothelial cell antibodies can further intensify vascular inflammation [30]. Genetic predisposition is also pertinent; in vitro research by Nickel et al. [3] demonstrated that *BMPR2* mutations, the predominant genetic cause of PAH [31], impair the tube formation capacity of retinal endothelial cells and modify their angiogenesis-related gene expression (e.g., elevated *CXCL10*, reduced *PLG*). Collectively, these processes could culminate in the structural changes underlying the observed reduction in VD on OCTA.

We observed that the FAZ area remained statistically unchanged while the surrounding FAZ FD was significantly diminished in the PAH group. This suggests that the perfusion within the critical perifoveal capillary network is compromised prior to the anatomical expansion of the avascular zone, positioning FAZ FD as a potentially more sensitive early indicator of macular ischemia.

Our detection of a significant reduction in global mean GCC thickness aligns with the significant thinning previously reported by Gu et al. [15]. However, the absence of significant changes in mean RNFL thickness may support a “vessels first” hypothesis of disease progression: retinal damage may begin with compromised capillary perfusion, with subsequent neuronal loss occurring as a secondary event, potentially before RNFL loss is detectable. This finding of significant global thinning indicates a generalized neurodegenerative process. Consequently, our study might have captured a stage of ocular damage in PAH where microvascular changes and GCC thinning are present, potentially preceding overt RNFL atrophy.

In contrast to the significant GCC thinning, we did not find significant differences in any of the macular retinal thickness subfields or in the optic disc area (Table 5). While our investigation centered on the retinal microvasculature, the literature delineates a broader spectrum of ocular involvement associated with PAH [32]. The choroid, characterized by high blood flow and sympathetic innervation [33], is susceptible to systemic hemodynamic fluctuations [34], but findings in PAH remain inconsistent. Some reports indicate increased subfoveal choroidal thickness in PAH patients [35], potentially attributable to acute venous congestion or the effects of frequently used PDE-5 inhibitors (e.g., sildenafil, tadalafil), which can augment choroidal blood flow [36]. Conversely, the cross-sectional analysis conducted by Zonenberg et al. [37] revealed significantly reduced choroidal thickness and volume in patients with precapillary pulmonary hypertension relative to a control group. This latter observation might be ascribed to chronic remodeling or atrophy secondary to long-standing disease or hypoxia. Furthermore, the same study identified no significant difference in the choroidal vascularity index and no correlation between choroidal parameters and most cardiological severity indices, suggesting potential limitations in the capacity of choroidal measurements to reflect systemic status accurately [37].

Establishing whether retinal microvascular alterations reflect systemic disease severity is crucial for determining their potential biomarker value. If significant associations do exist between diminished VD or other retinal parameters (e.g., tortuosity) and established clinical severity indicators, the potential utility of OCTA as a prognostic instrument will be increased. Retinal vascular imaging holds potential for monitoring disease development and/or progression. The concept of a “lung–eye axis” supports this potential [3]. However, the absence of a correlation between choroidal parameters necessitates caution, implying that not all ocular parameters may serve as direct surrogates for systemic hemodynamics. Retinal VD and perhaps tortuosity appear to be more promising. The combination of poor prognosis and systemic involvement underscores the critical need for reliable, noninvasive biomarkers for early detection, risk stratification, and monitoring of disease progression.

There are several limitations of the present study. The cross-sectional design of this study precludes the inference of causality. Its single-center origin and sample size may restrict the generalizability. Another limitation is the potential influence of PAH-specific vasoactive therapies on retinal microvascular parameters; as most patients were receiving standard treatment, this constitutes a confounding factor stemming from both disease modulation and direct drug effects. Additionally, the study lacks correlation analyses between OCTA parameters and cardiopulmonary severity indices due to the absence of synchronized hemodynamic and functional data. Future longitudinal studies are needed to clarify the biomarker value of OCTA in pulmonary arterial hypertension. Strengths of the study include the utilization of a well-matched control group, the focus on a homogeneous cohort of patients with group 1 PAH, and adherence to stringent image quality standards based on SSI values of ≥6. To the best of our knowledge, the present study includes one of the largest cohorts among original research articles to date that employ OCTA to investigate similar patient groups.

## 5. Conclusion

This study has provided compelling quantitative evidence, derived noninvasively via OCTA, to demonstrate that PAH is associated with significant and widespread microvascular rarefaction affecting both the SCP and DCP, alongside reduced perifoveal perfusion indicated by lower FAZ FD values. These findings strongly corroborate the systemic nature of PAH vasculopathy, clearly extending beyond the pulmonary circulation. Although significant thinning was detected in the global mean GCC, the pronounced and statistically robust vascular changes emphatically highlight the potential utility of OCTA as a noninvasive tool for assessing and potentially detecting systemic microvascular involvement in patients with PAH. To confirm these cross-sectional findings and establish OCTA as a prognostic biomarker, future prospective longitudinal investigations incorporating comprehensive hemodynamic data are essential.

## Figures and Tables

**Table 1:** Demographic and clinical characteristics of the study population.

Parameter	PAH group (n=40)	Healthy control group (n=38)	p-value
Age (years), Mean ± SD	55.55 ± 16.78	50.42 ± 17.05	0.180
Sex, n (%) Female / Male	32 (80.0%) / 8 (20.0%)	23 (60.5%) / 15 (39.5%)	0.059
BCVA (Snellen), Mean ± SD	0.75 ± 0.25	0.82 ± 0.18	0.165
IOP (mmHg), Mean ± SD	14.78 ± 2.25	14.50 ± 2.10	0.582
SE (Diopters), Mean ± SD	+0.27 ± 1.30	+0.25 ± 1.15	0.941

PAH: Pulmonary arterial hypertension; SD: standard deviation; BCVA: best-corrected visual acuity; IOP: intraocular pressure; SE: spherical equivalent.

**Table 2:** Superficial capillary plexus vascular density (%) in pulmonary arterial hypertension (PAH) patients and the healthy control group.

Parameter	PAH group (n=40) mean (±SD)	Healthy control group (n=38) mean (±SD)	p-value
Superior quadrant	45.75 ± 4.65	47.65 ± 2.10	**0.006**
Inferior quadrant	45.64 ± 4.92	47.49 ± 1.74	**0.020**
Whole image	45.73 ± 4.61	47.59 ± 1.83	**0.004**
ETDRS grid	45.67 ± 4.47	47.46 ± 1.73	**0.005**

SD: Standard deviation; ETDRS: Early Treatment Diabetic Retinopathy Study. Values of p < 0.05 are indicated in bold.

**Table 3:** Deep capillary plexus vascular density (%) in pulmonary arterial hypertension (PAH) patients and the healthy control group.

Parameter	PAH group (n=40) mean (±SD)	Healthy control group (n=38) mean (±SD)	p-value
Superior quadrant	48.31 ± 6.27	50.79 ± 5.79	**< 0.001**
Inferior quadrant	47.30 ± 7.63	49.75 ± 5.29	0.074
Whole image	47.89 ± 6.69	50.28 ± 5.39	**0.006**
ETDRS grid	48.80 ± 6.95	50.72 ± 5.66	**0.008**

SD: Standard deviation; ETDRS: Early Treatment Diabetic Retinopathy Study. Values of p < 0.05 are indicated in bold.

**Table 4:** Foveal avascular zone (FAZ) metrics in pulmonary arterial hypertension (PAH) patients and the healthy control group.

Parameter	Unit	PAH group (n=40) mean (±SD)	Healthy control group (n=38) mean (±SD)	p-value
Area	mm^2^	0.309 ± 0.324	0.286 ± 0.139	0.779
Perimeter	mm	2.23 ± 1.37	2.04 ± 0.46	0.972
Flow density	%	45.42 ± 6.06	49.85 ± 6.81	**0.001**

SD: Standard deviation. Values of p < 0.05 are indicated in bold.

**Table 5:** Retinal and neuroretinal thickness measurements (μm) in pulmonary arterial hypertension (PAH) patients and the healthy control group.

Parameter	PAH group (n=40) mean (±SD)	Healthy control group (n=38) mean (±SD)	p-value
Fovea	253.5 ± 20.1	255.6 ± 21.2	0.749
Parafovea	313.8 ± 19.5	320.4 ± 14.7	0.063
Perifovea	282.7 ± 18.0	280.0 ± 10.0	0.980
ETDRS grid	287.9 ± 13.6	287.5 ± 9.5	0.972
Mean GCC	108.7 ± 11.64	115.1 ± 6.65	**0.006**
Mean RNFL	93.90 ± 10.69	93.2 ± 9.94	0.267
Disc area	1.90 ± 0.46	1.89 ± 0.38	0.686

SD: Standard deviation; ETDRS: Early Treatment Diabetic Retinopathy Study; GCC: ganglion cell complex; RNFL: retinal nerve fiber layer. Values of p < 0.05 are indicated in bold.
